# Grafting with Rabbit Skin

**Published:** 1872-10

**Authors:** 


### Grafting with Rabbit Skin.

There have been reported to the Paris Academy of Sciences three cases of ulcers in the human subject healed by grafting upon them cutaneous particles taken from the rabbit. M. Larry was so pleased with the idea that he proposed entering the Report for a prize. Whether the new surface produced a growth of rabbit's fur, we are not informed. But why not? And if so, why not put the new practice to profit by substituting for the rabbit the Angora goat or some other variety which will produce a valuable wool? Baron Munchausen did something of this kind, if our memory serves us, with his horse. There are some thousands of vagabonds and convicts in California, who might be made valuable to the State, or at least who would pay for their keeping, if, by the aid of delicate surgery, they were set to raising wool.—*Chicago Medical Examiner.*

